# Author Correction: Accelerated western European heatwave trends linked to more-persistent double jets over Eurasia

**DOI:** 10.1038/s41467-023-40267-0

**Published:** 2023-07-28

**Authors:** Efi Rousi, Kai Kornhuber, Goratz Beobide-Arsuaga, Fei Luo, Dim Coumou

**Affiliations:** 1 Potsdam Institute of Climate Impact Research (PIK), Member of the Leibniz Association, Potsdam, Germany; 2 Earth Institute, Columbia University, New York, NY USA; 3 Lamont-Doherty Earth Observatory, Columbia University, New York, NY USA; 4 International Max Planck Research School on Earth System Modelling, Hamburg, Germany; 5 Institute of Oceanography, Center for Earth System Sustainability, Universität Hamburg, Hamburg, Germany; 6 Institute for Environmental Studies, Vrije Universiteit Amsterdam, Amsterdam, Netherlands; 7 Royal Netherlands Meteorological Institute (KNMI), De Bilt, Netherlands

**Keywords:** Atmospheric dynamics, Climate-change impacts, Environmental impact

Correction to: *Nature Communications* 10.1038/s41467-022-31432-y, published online 04 July 2022

The original version of this Article contained an error in Figure 5c, and Supplementary Figures 6c, 7c, and 8c, in which the y-axis was incorrectly plotted up to a lower maximum value and resulted in one data point missing. In the corrected versions, the y-axis has been extended to higher values and include the missing data point. This has been corrected in both the PDF and HTML versions of the Article. The HTML has been updated to include a corrected version of the Supplementary Information.

The original version of this Article contained an error in the Author Contribution section, which incorrectly read ‘E.R., D.C., K.K., G.B.-.A. and F.C. contributed to the interpretation of the results and the final form of the manuscript.’ The correct version states ‘E.R., D.C., K.K., G.B.-.A. and F.L. contributed to the interpretation of the results and the final form of the manuscript.’ This has been corrected in both the PDF and HTML versions of the Article.

## Supplementary information

Updated Supplementary Information

