# Supplementary material for: Accelerated western European heatwave trends linked to more-persistent double jets over Eurasia
Source: Nat Commun. 2022 Jul 4;13:3851. doi: 10.1038/s41467-022-31432-y (PMC9253148; doi:10.1038/s41467-022-31432-y)
Supplement: Supplementary file 1 — Supplementary Information [file 41467_2022_31432_MOESM1_ESM.pdf]

## Supplementary Information

for

# Accelerated western European heatwave trends linked to more-persistent double jets over Eurasia

Efi Rousi[1*] (rousi@pik-potsdam.de), Kai Kornhuber[2,3,1], Goratz Beobide-Arsuaga[4,5], Fei Luo[6,7], Dim Coumou[6,7,1]

1 Potsdam Institute of Climate Impact Research (PIK), Member of the Leibniz Association, Potsdam, Germany

2 Earth Institute, Columbia University, New York, US;

3 Lamont-Doherty Earth Observatory, Columbia University, New York, US

4 International Max Planck Research School on Earth System Modelling, Hamburg, Germany

5 Institute of Oceanography, Center for Earth System Sustainability, Universität Hamburg, Hamburg, Germany

6 Institute for Environmental Studies, Vrije Universiteit Amsterdam, Amsterdam, Netherlands

7 Royal Netherlands Meteorological Institute (KNMI), De Bilt, Netherlands

**Table S1.** List of the 20 longest double jet events in the studied period with their start date, end date and duration.

| Start date | Duration (in days) |
|---|---|
| 21.07.2003 | 29 |
| 06.07.2001 | 26 |
| 23.07.1994 | 25 |
| 19.07.2013 | 25 |
| 26.07.1995 | 23 |
| 04.07.2018 | 22 |
| 07.07.1988 | 21 |
| 10.07.2000 | 20 |
| 11.07.2006 | 20 |
| 11.07.2010 | 20 |
| 02.07.2013 | 16 |
| 17.07.2008 | 13 |
| 24.07.1980 | 12 |
| 01.07.1994 | 12 |
| 07.08.1990 | 11 |
| 15.07.1993 | 11 |
| 31.07.2007 | 11 |
| 12.07.2009 | 11 |
| 13.08.2009 | 11 |
| 15.08.2017 | 11 |

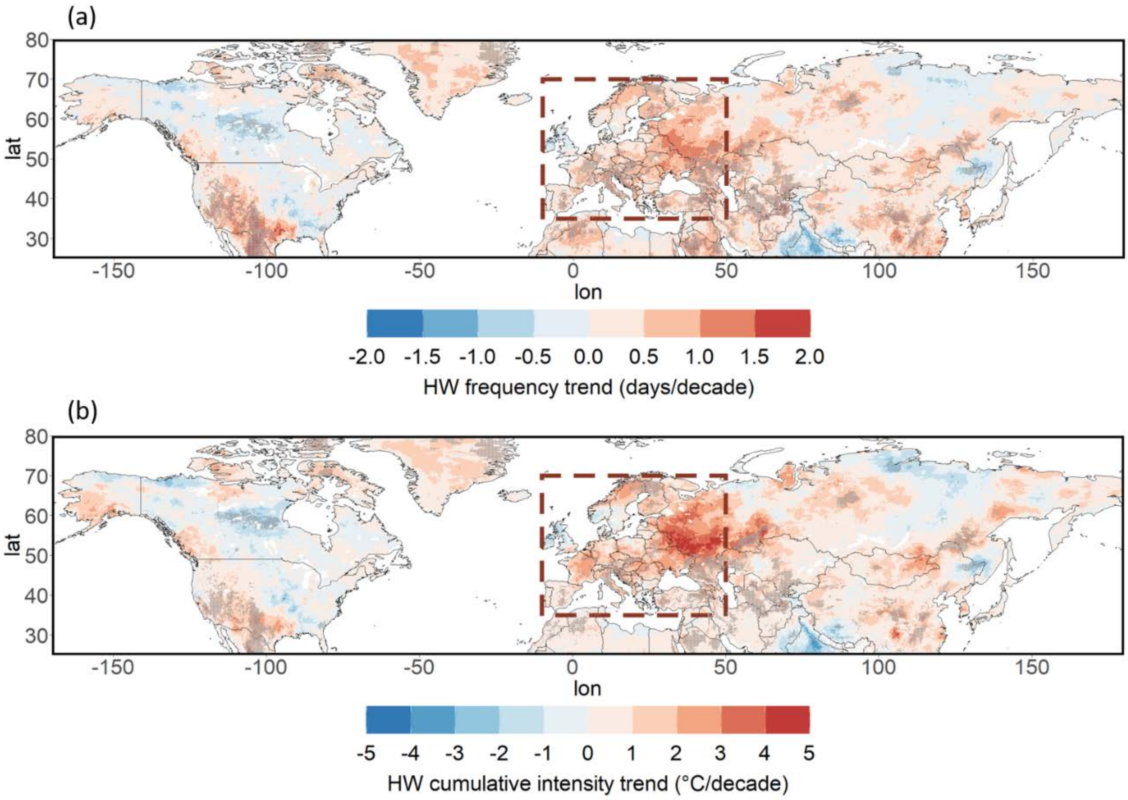


**Figure S1. Increasing heatwave (≥ 6 consecutive days) trends over the midlatitudes and Europe.** **(a)** Decadal trends in heatwave (heatwaves are defined as at least 6 consecutive days of threshold exceedance) frequency (days/decade) and **(b)** heatwave cumulative intensity (°C/decade) for July-August 1979-2020. Statistically significant trends ($p<0.05$) are marked with grey dots. The dashed red box contains the extended European region as discussed in this paper (35-70°N and 10oW-50°E).

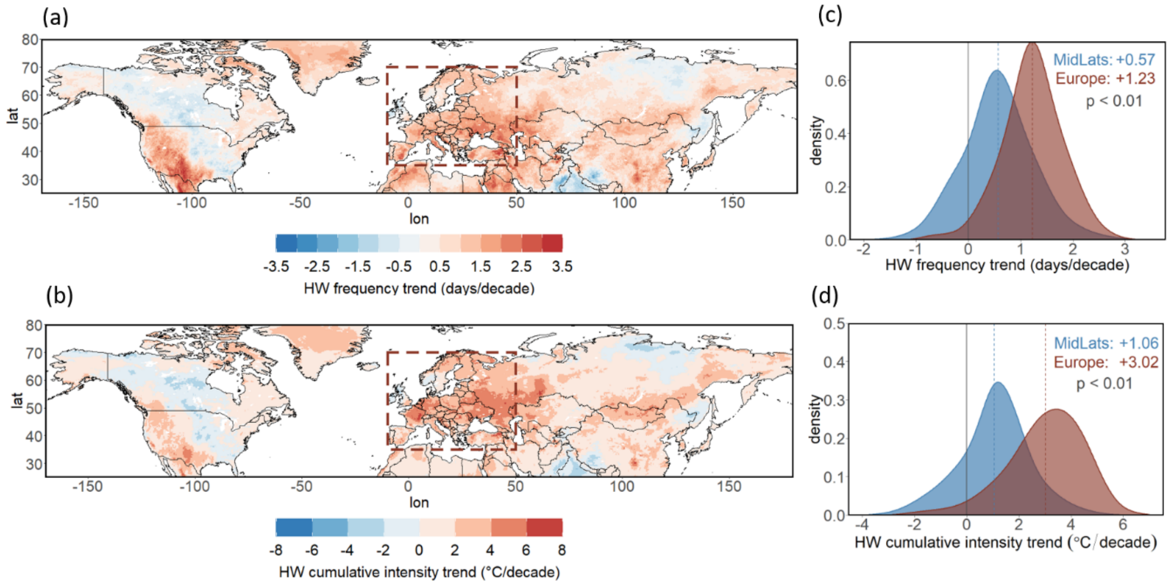


**Figure S2. Increasing heatwave (≥ 3 consecutive days) trends over the midlatitudes and Europe**. **(a)** Decadal trends in heatwave (heatwaves are defined as at least 3 consecutive days of threshold exceedance) frequency (days/decade) and **(b)** heatwave cumulative intensity (°C/decade) for July-August 1979-2020. **(c)** Probability density distributions of decadal trends of heatwave frequency of all land grid points for Europe (in dark red, as the region included in the dashed box of panels a and b: 35-70°N and 10oW-50°E) and the midlatitudes (20-70°N) excluding Europe (in blue) and **(d)** probability density distributions of decadal trends of heatwave cumulative intensity. The mean trend for each distribution is shown with dashed vertical lines and provided on the top right of the panels. The continuous vertical lines correspond to 0 (i.e. no trend). The two distributions were compared for each case with a Kolmogorov-Smirnov test (p-values shown on the center right).

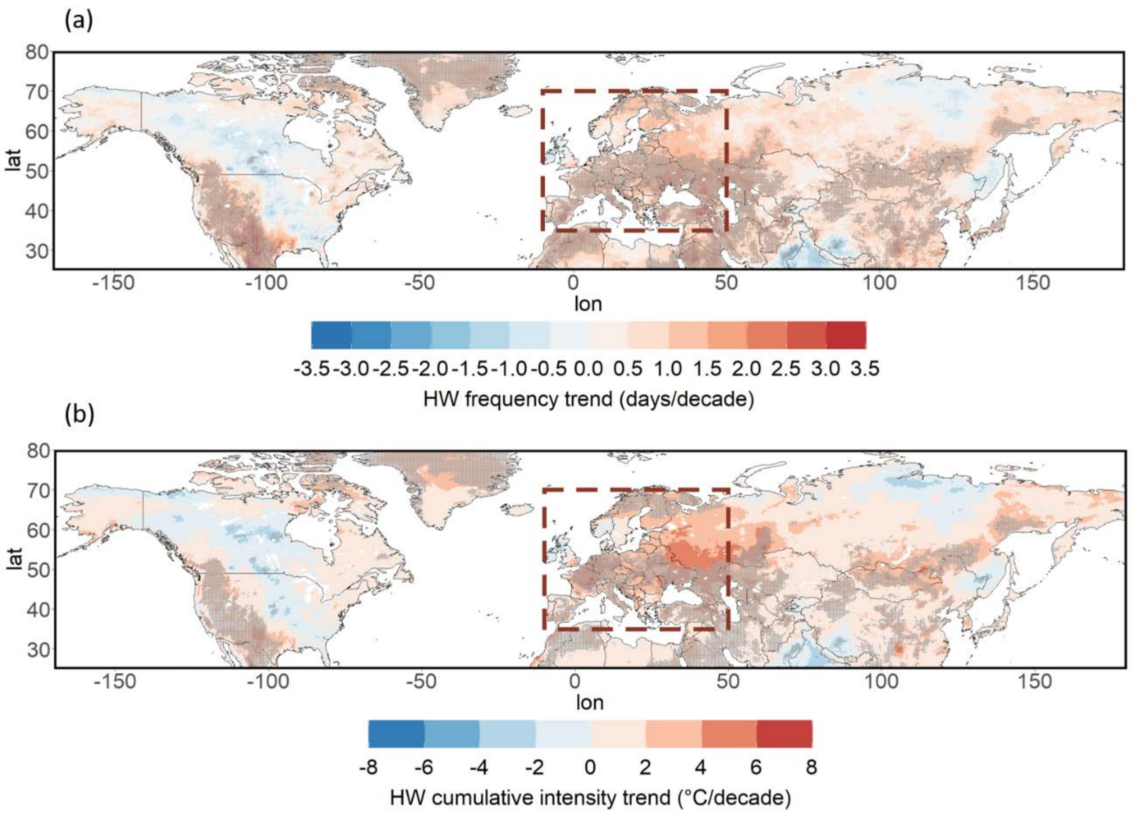


**Figure S3.** As in Figure S2a and b but with statistically significant trends ($p<0.05$) marked with grey dots.

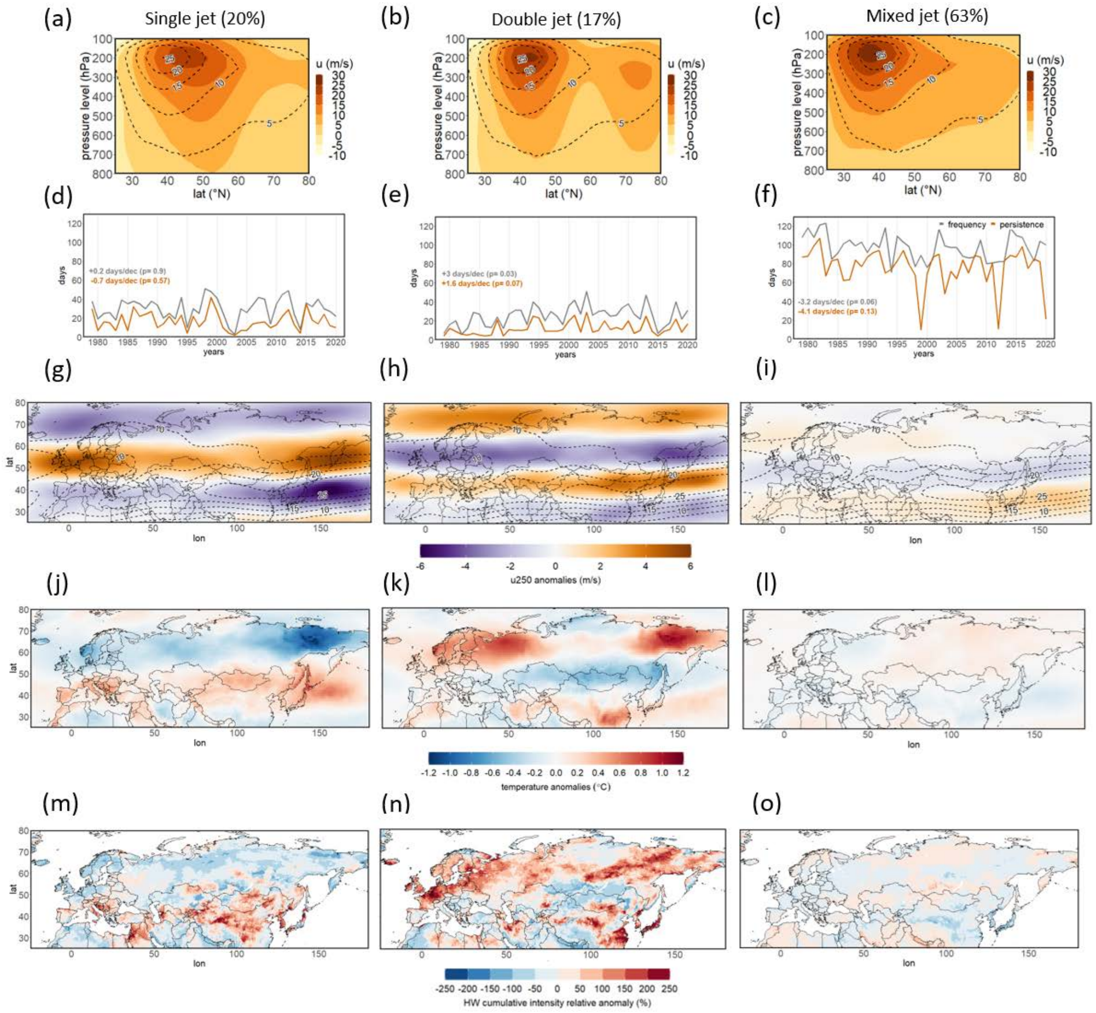


**Figure S4. Jet stream states and surface temperature for the extended summer period May-September (MJJAS). (a-c)** Composites of the vertical profile of the zonal (averaged over the Eurasian domain) mean zonal wind (u, shading) with frequency of occurrence provided in parenthesis. The climatological mean of the zonal mean zonal wind for the whole period is plotted with dashed contours (plotted from 5 to 20m/s every 5m/s). **(d-f)** Frequency (grey line) and maximum persistence (orange line) of each cluster per year. The decadal linear trend and respective p-values are given for both timeseries on the top left of the panels. **(g-i)** Anomaly composites of (linearly detrended) zonal wind at the 250hPa pressure level (u250) for each cluster (shading). The climatological mean of the zonal wind at 250hPa is plotted with dashed contours (plotted from 5 to 25m/s every 5m/s). **(j-l)** Anomaly composites of (linearly detrended) mean surface temperature for each cluster. Anomalies in both cases are calculated with respect to daily climatology (to remove the seasonal cycle). **(m-o)** Composites of heatwave cumulative intensity (calculated after having removed the Tmax mean midlatitude-land trend

from each grid point) shown as relative anomaly (%) compared to the climatology. All figures refer to the months of May-September of the period 1979-2020.

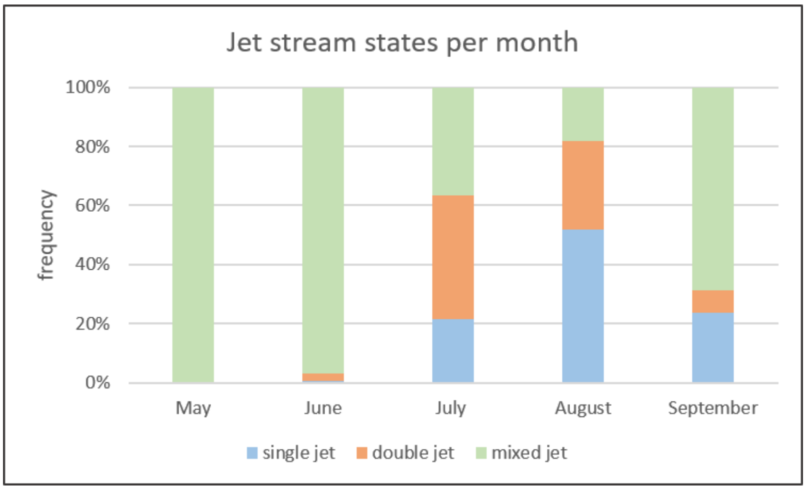


**Figure S5. Frequency of the three jet stream states for each of the months May-September.**

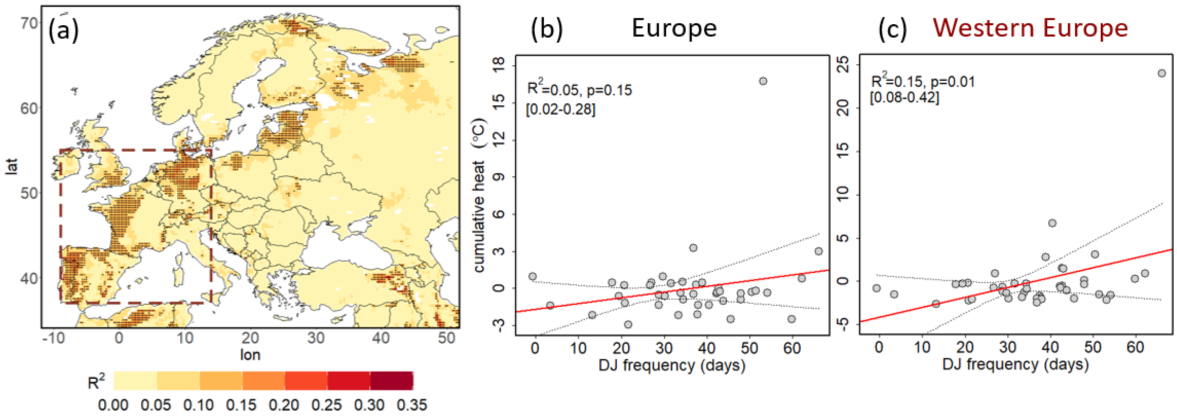


**Figure S6. Explained variance of heatwave cumulative intensity by double jet frequency. (a)** Explained variance ($R^2$) per grid point of heatwave cumulative intensity based on linear regression on double jet frequency. Statistically significant coefficients ($p<0.05$) are marked with black dots. **(b)** Scatter plots of heatwave cumulative intensity anomalies aggregated over all land grid points of the extended European domain (as seen in panel a and in the brown dashed box of Figure 1a and b) and double jet frequency. A linear fit (in red) and its confidence interval (dashed lines for the 5th and 95$^{th}$ percentiles obtained from 1000 bootstraps), $R^2$ (with 5th and 95th interval percentiles obtained from 1000 bootstraps in brackets), and p-values are shown on the top left of each plot. **(c)** As for (b) but with heatwave cumulative intensity aggregated only over land grid points with statistically significant

coefficients in western Europe (dotted points in panel a within the region included in the dashed red box: 37-55˚N and 9˚W-14˚E). The linear trend of the timeseries was removed before the regression was applied in all cases.

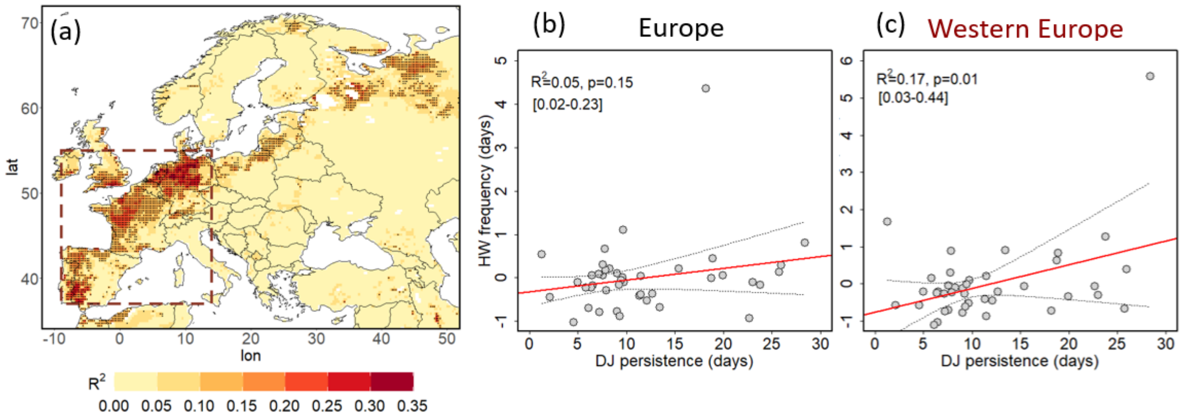


**Figure S7.** As in Figure S6 but for regression of heatwave frequency on double jet persistence.

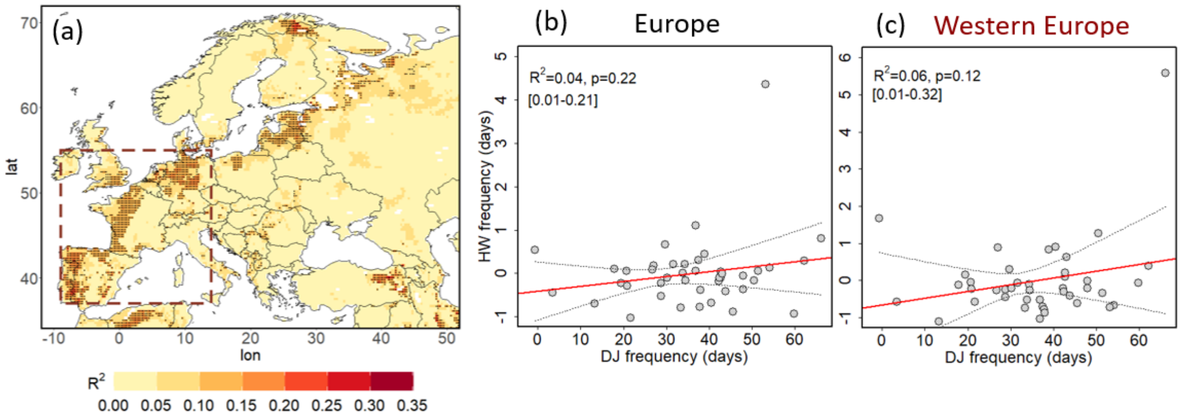


**Figure S8.** As in Figure S6 but for regression of heatwave frequency on double jet frequency.
